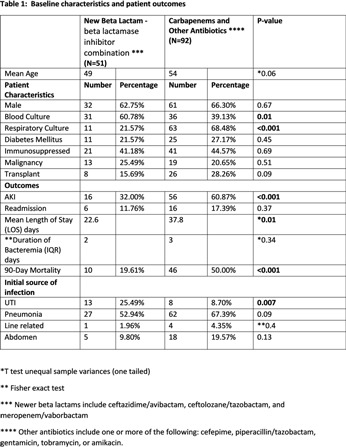# Evaluation of Ceftolozane/Tazobactam, Ceftazidime/Avibactam and Meropenem/Vaborbactam Against Multidrug-Resistant (MDR) *Pseudomonas*

**DOI:** 10.1017/ash.2021.134

**Published:** 2021-07-29

**Authors:** Haider Shamsulddin, Jeffrey Lin, Julie Ribes, Thein Myint

## Abstract

**Background:** Data on the patient outcomes for newer β-lactam–β-lactamase inhibitor (BLBI) drugs compared to carbapenem-containing combination antibiotics for multidrug-resistant (MDR)–*Pseudomonas aeruginosa* infections are limited. **Methods:** This retrospective, case–control observational study was based on chart review of the patients managed at the University of Kentucky. **Results:** In total, 143 patients with MDRO *Pseudomonas aeruginosa* infections were identified and divided into 2 groups: 1 group received newer BLBI combinations with or without aminoglycosides or polymyxins, for at least 72 hours, and the control group received carbapenem containing combination antibiotics or other antibiotics. Baseline characteristics and patient outcomes are shown in Table 1. **Discussion:** The newer BLBI combinations group consisted of 60.8% MDR *Pseudomonas* bacteremia, whereas the control group had 68.4% of MDR *Pseudomonas* respiratory cultures. Overall, the use of newer BLBI combinations such as ceftazidime/avibactam, ceftolozane/tazobactam, and meropenem/vaborbactam was associated with lower rates of acute kidney injury (AKI), shorter LOS, and lower mortality rates compared to the control group, and these differences were statistically significant. Because the 2 populations of patient differed significantly based on the site of infection (sepsis vs pneumonia), the data were reanalyzed to evaluate the impact of therapy on the occurrence of AKI, LOS, and mortality based on the site of infection. Only those patients with sepsis who received the newer combination drugs had significantly better rates of AKI, lower LOS, and had lower rates of mortality. The 2 treatment arms were not statistically different when comparing patients with pneumonia. Additionally, the use of these new combination therapies did not make a difference regarding readmission rates or duration of bacteremia for the patients included in the study.

**Funding:** No

**Disclosures:** None

**Table 1.**
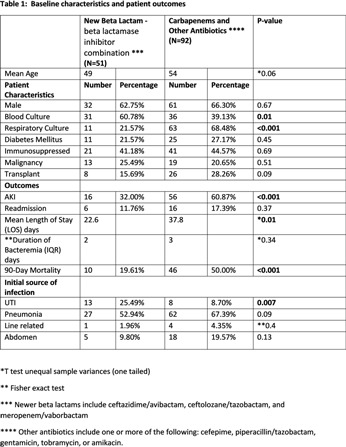

**Table 2.**